# Effectiveness of physically ablative and pharmacological treatments for anal condyloma in HIV-infected men

**DOI:** 10.1371/journal.pone.0199033

**Published:** 2018-08-01

**Authors:** Sandra Vela, Sebastian Videla, Arelly Ornelas, Boris Revollo, Bonaventura Clotet, Guillem Sirera, Marta Piñol, Francesc García-Cuyás

**Affiliations:** 1 Department of Surgery, University Hospital Germans Trias i Pujol, Badalona, Catalonia, Spain; 2 Lluita Contra La SIDA Foundation, University Hospital Germans Trias i Pujol, Badalona, Catalonia, Spain; 3 Department of Clinical Pharmacology, University Hospital Bellvitge / IDIBELL / Barcelona University, Hospitalet de Llobregat, Barcelona, Catalonia, Spain; 4 HIV Clinical Unit, Department of Medicine, University Hospital Germans Trias i Pujol, Badalona, Catalonia, Spain; 5 Retrovirology Laboratory IrsiCaixa Foundation, Badalona, Catalonia, Spain; Rudjer Boskovic Institute, CROATIA

## Abstract

**Background:**

There is limited information on the effectiveness of available treatments for anal condyloma acuminata in HIV-1-infected men.

**Aim:**

To provide data on the effectiveness of electrosurgical excision, infrared coagulation and pharmacological (imiquimod) treatments for anal condyloma acuminata (peri-anal and/or intra-anal) in HIV-1-infected men based on authors’ practice.

**Methods:**

Single-center, retrospective descriptive analysis of HIV-1-infected men, 18 years or older treated for anal condyloma acuminata. Standard treatments were offered: electrosurgery excision, infrared coagulation and topical imiquimod. Effectiveness was evaluated by the recurrence rate at 1 year after treatment. Recurrence was defined as any anal condyloma acuminata diagnosed after 3 months of condyloma-free survival post-treatment. Anal cytology and human-papillomavirus-infection (HPV) was assessed.

**Results:**

Between January 2005 and May 2009, 101 men were treated for anal condyloma acuminata: 65 (64%) with electrosurgery, 27 (27%) with infrared coagulation and 9 (9%) with imiquimod. At 1 year after treatment, the cumulative recurrence rate was 8% (4/65, 95%CI: 2–15%) with electrosurgery excision, 11% (3/27, 95%CI: 4–28%) with infrared coagulation and 11% (1/9, 95%CI: 2–44%) with imiquimod treatment. No predictive factors were associated with recurrence.

Anal HPV-6 or HPV-11 was detectable in 98 (97%) patients and all had high-risk HPV genotypes, and 89 (88%) patients had abnormal anal canal cytology. Limitations: this was a retrospective descriptive analysis; limited to a single center; it cannot know if the recurrence is related to new infection.

**Conclusion:**

Recurrence of anal condyloma after any treatment was common. Abnormal anal cytology and high-risk HPV-infection were highly prevalent in this population, therefore at high-risk of anal cancer, and warrants careful follow-up.

## Introduction

Anal condyloma acuminata (CA) are frequently associated with human papillomavirus (HPV) types 6 and 11 [1,2]. CA may be located peri-anally or intra-anally and patients commonly present for medical treatment due to feeling ‘bumps’ when washing or more infrequently after findings on routine medical examinations such as colonoscopy, or more rarely with symptoms such as itch, wetness or pain. Global incidence of anogenital warts ranges from 160 to 289 cases per 100 000 person-years [3,4]. The histology of anogenital warts typically shows benign characteristics, although intraepithelial or invasive squamous cell carcinomas can coexist [5].

There is limited scientific literature available on the effectiveness and safety of treatments used in clinical practice for anal CA [6]. Moreover, there is no consensus on the best treatment to manage these lesions. In clinical practice, there are various treatment modalities including physical ablation [electrosurgery excision, cryotherapy, infrared coagulation (IRC), laser ablation], and pharmacological treatments (imiquimod, podophyllotoxin, tricloracetic acid, sinecatechins) [7–9]. Generally, the election of the treatment depends on localization, size, number of the CAs and on the doctor’s experience. For example, physically ablative treatments are used at peri- and intra-anal area, meanwhile pharmacological treatments are indicated when the lesions exclusively affect the perianal area. A high regression rate in the first year after diagnosis of genital warts can be common among HIV-1-infected women (on 60%) and in general population (80%) [10]. However, despite this regression rate, the main challenge in clinical practice for any treatment is the high percentage of recurrence, after a long period of follow-up, independent of the type of treatment used [7,11–13]. Albeit these treatments can result in resolution of the wart, removing the lesion is not synonymous with eradicating the HPV infection, and this may explain the high recurrence rate, regardless of the treatment modality employed.

Therefore, the aim of this study was simple, to provide data on the effectiveness and safety of electrosurgery excision, IRC and imiquimod treatments for anal CA in HIV-1-infected men, based on authors’ clinical practice.

It is noteworthy that this study does not aim to compare the effectiveness among treatments, and is a descriptive analysis. Likewise, the results of the present study are complementary to data of previous published works: data on the prevalence of anal CA [14] from the CARH·MEN cohort [15].

## Patients and methods

### Study design

The study was a single-center, retrospective analysis using data from a prospectively compiled database of outpatients included in CARH·MEN cohort [15] and to whom anal CA was diagnosed. In brief, CARH·MEN cohort is a screening program for detection and treatment of anal intraepithelial neoplasia.

The study was approved by the Hospital Investigational Review Board (University Hospital Germans Trias i Pujol, Badalona, Catalonia, Spain). Data confidentiality was ensured according to Spanish legislation on the protection of personal data (LOPD 15/1999).

The starting point of this study was January 1^st^ 2005, when the Clinical Proctology HIV Section was created and CARH·MEN cohort started. Hence, this study embraces the first 10 years of our outpatients Clinical Proctology HIV Section of the Germans Trias i Pujol University Hospital (Badalona, Spain).

### Study population

The patients included had to fulfill the following criteria: age ≥18 years, men from CARH·MEN cohort at first visit, no medical history of anal CAs, a visual diagnosis of a CA at peri-anal and/or intra-anal (including at high-resolution anoscopy (HRA), treated in our hospital and with at least a year of follow-up after treatment.

The period of follow-up was defined as the time between the baseline (corresponding to baseline visit of CARH·MEN cohort and when the CA was diagnosed) and last available documented visit (up to the diagnosis of anal CA recurrence or up to the last documented visit).

The following data were gathered at baseline visit (CA diagnosis): date of birth, date of HIV diagnosis (time of known HIV), date of the visit, CD4 T cell count (the most recent value before the wart diagnosis visit), nadir CD4 T count, plasma HIV-1 viral load, antiretroviral therapy (ART) before inclusion (Yes/No), date of starting the ART (time on ART), anal canal cytology (normal, atypical squamous cells of unknown significance [ASCUS], low-grade squamous intraepithelial lesion [LSIL], or high-grade squamous intraepithelial lesion [HSIL]) results, HPV DNA testing and typing at anal canal sample. The following data during the follow-up was also gathered: type of treatment for wart performed, recurrence (YES/NO), date of recurrence and adverse events reported by the patients and related to the procedures.

### Anal CA diagnosis

The diagnosis was based on visual inspection or HRA. Each visit of CARH·MEN cohort includes a clinical examination (visual inspection) including a digital rectal examination and to obtain a sample from the anal canal for cytological examination. If the cytological result at baseline was abnormal (ASCUS, LSIL, or HSIL) a HRA was performed.

### Treatments available

A guidance clinical protocol for the treatment of anal CA, based on the number of condyloma, size of affected area (diameter, cm^2^) location and characteristics of the patients, was available in the Clinical Proctology HIV Clinic. A brief summary of the treatments offered in our proctology unit:

- Electrosurgery excision under anesthesia. Electrosurgery treatment was proposed when more than 1 CA greater than 0.5 cm^2^ with each one affecting the peri-anal and/or intra-anal areas (circumferential or near-circumferential area affected), or to patients with more than 5 small CAs smaller than 0.5 cm^2^ each one localized at peri-anal and/or intra-anal areas, or when at least a single CA greater than 1 cm^2^ was diagnosed, or when the patients had a poor tolerance to HRA. This treatment consists on the excision of all visible lesions by electrocautery (electric scalpel). In some cases, several interventions could be required to eliminate all lesions to avoid the risk of anal stenosis.
- Ablation with IRC (Redfield IRC-2100; Redfield-Corporation, Rochelle Park, New Jersey, USA). IRC was offered to patients with a single CA of anal canal smaller than 1 cm^2^, or to patients with up to 5 small CAs smaller than 0.5 cm^2^ each one localized at peri-anal and/or intra-anal areas or when a surgical excision could be very traumatic according the treating surgeon. The anal condylomata were treated with in-office IRC ablation in the same facilities of HIV proctology service. Before performing ablation with IRC directed by HRA, anal condyloma was identified and local anesthesia (mepivacaine, Scandinibsa^®^, INIBSA-HOSPITAL, S.L.U., Lliçà de Vall, Barcelona, Spain) was used. IRC delivers short pulses of a narrow beam of visible infrared light through a small contact tip applicator that was applied directly to the condyloma. It results in thermal coagulation and tissue necrosis. The scab was removed, and the process was repeated with 1.5-second pulses until the submucosal vessels were coagulated;
- Pharmacologic treatment. Topical imiquimod (Aldara 5% cream^®^, Meda-AB, Solna, Sweden) three times per week, at night before going to the bed, and a minimum of 12 weeks or until the lesions disappear was offered to patients with peri-anal small wart (lower than 0.5 cm^2^).

The abovementioned treatments (electrosurgery excision, IRC and imiquimod) were managed for clinicians from the Clinical Proctology HIV Clinic. Treatment modality was selected based on clinician recommendation and patient preference. That is to say, the different treatments available were explained to the patients, the doctor in charge based on his/her experience advised, but the decision to select one was agreed with the patient. After being treated, the patients were reviewed every 3 months in the first year (including HRA at first control visit), and annually thereafter.

### Anal canal samples: Cytology and HPV detection

#### Anal canal cytological procedure

Anal canal sample for cytological examination was obtained, according previously described [16]. This sample was used to carry out the cytology analysis (Papanicolaou test). Anal canal cytological changes were classified according to the Bethesda System as normal, ASCUS, LSIL and HSIL.

#### HPV detection

Detection of HPV Infection was performed in the anal cytological sample according previously described [16]. HPV was detected and typed using the F-HPV typing™ kit (Molgentix, Spain) for HPV-6,-11,-16,-18,-31,-33,-35,-39,-45,-51,-52,-56,-58,-59,-and -68.

### Statistical analysis

#### Sample size

The sample size was defined as the number of patients from CARH·MEN cohort who fulfilled the inclusion criteria.

#### Definitions

Recurrence of anal condyloma was defined as the reappearance of any evidence of anal condyloma after 3 months of condyloma-free survival post-treatment, assessed by physical examination for perianal-CA and by HRA for intra-anal-CA. The effectiveness of treatments was assessed by means of the recurrence rate.

#### Statistical procedures

A descriptive analysis was performed for baseline population characteristics. Recurrence (number of patients) was estimated and its 95% confidence intervals (95%CI) was calculated.

Bivariate and multivariate logistic analyses and Cox proportional hazard regression models were performed, when appropriate, to determine potential factors associated with the recurrence. Recurrence rates were also calculated based on person-time denominator (100 person-years).

The association between potential explanatory variables and anal condyloma was tested in 2 steps. First, a bivariate regression model was performed to obtain an indication of the relevance of the explanatory variables in the risk of HIV-1 infection. Second, factors showing a P value less than 0.3 were used to perform a multiple regression model using a stepwise selection of variables. Hazard ratios (HRs) for incidence were estimated as well as their corresponding 95%CI. A P value of 0.05 or less was considered statistically significant. Data analysis was performed using SPSS version 15.0 (SPSS Inc. Chicago, IL) and R version 3.3.1 (2016-06-21).

## Results

### Patient characteristics

One hundred and fifty-seven patients from CARH·MEN cohort were diagnosed with peri-anal and/or intra-anal condyloma [14,15]. Of them, 103 were treated in our hospital, and had at least 1 year of follow-up after the wart treatment. Given that the descriptive nature of this work, two patients treated with cryotherapy in our hospital but out of HIV facilities were also included. This treatment was managed in dermatology department of our hospital. The two patients treated with cryotherapy presented with a peri-anal small wart (lower than 0.5 cm^2^). This treatment consists on the application of liquid nitrogen to freeze and destroy the CA as well as the immediate surrounds area. Therefore, 65 (63%) patients were treated with electrosurgery excision, 27 (26%) with IRC, 9 (9%) with pharmacological treatment and 2 (2%) with cryotherapy. Table 1 shows the baseline characteristics. The group of patients treated with IRC were slightly older, with a lower nadir CD4 and with greater time of HIV-1-infection with respect to other treatments groups.

**Table 1:** Baseline characteristic.

Baseline characteristic		All n = 103	Electrosurgery excision n = 65	Infrared coagulation n = 27	Pharmacologic n = 9	Cryotherapy n = 2
Age	median (IQR)	38.8 (32.4–44.2)	38.4 (31.0–42.5)	42.3 (38.7–48.6)	30.3 (26.1–43.3)	38.2 (37.6–38.8)
Sexual behavior:						
MSM	n (%)	91 (88)	59 (91)	24 (89)	6 (67)	2 (100)
MSW	n (%)	12 (12)	6 (9)	3 (11)	3 (33)	—
Time of known HIV (years)	median (IQR)	6.9 (0.6–14.2)	5.7 (0.5–13.6)	10.2 (1.1–17.4)	2.5 (0.2–13.5)	8.2 (0.4–15.9)
ART (yes)	n (%)	73 (71)	47 (72)	19 (70)	6 (67)	1 (50)
Time on ART HIV (years)	median (IQR)	7.3 (0.0–9.9)	5.1 (0.3–9.6)	9.1 (5.4–10.6)	2.7 (0.1–8.6)	9.9 (9.8–10.0)
HIV-1 RNA plasma load (copies/mL)	median (IQR)	40 (40–40)	40 (40–40)	40 (40–80)	40 (40–142)	350020 (40–700000)
HIV plasma load, <80 copies/mL	n (%)	91 (88.3)	62(95.4)	21 (77.8)	7 (77.8)	1 (50)
CD4 nadir cells/uL	median (IQR)	269 (121–410)	282 (155–415)	204 (91–299)	316 (34–579)	346 (324–368)
CD4 nadir cells/uL (<200) (yes)	n (%)	37 (36)	21 (32)	13 (48)	3 (33)	0 (0)
CD4 cells/uL	median (IQR)	705 (482–900)	753 (499–953)	548 (426–731)	612 (278–1162)	1269 (880–1657)
CD4 cells/uL (<200) (yes)	n (%)	6 (6)	2 (3)	2 (7)	2 (22)	0 (0)

MSM: men who have sex with men; MSW: men who have sex with women; ART: antiretroviral therapy; IQR: interquartile range.

### Effectiveness of treatments for CA

All patients achieved a primary clearance at 3 months after treatment, no recurrence (assessed by physical examination for peri-CA and by standard or HRA for intra-anal CAs). The cumulative recurrence rate at 1 year after treatment was 8% (4/65, 95%CI: 2–15%) with electrosurgery excision, 11% (3/27, 95%CI: 4–28%) with infrared and 11% (1/9, 95%CI: 2–44%) with pharmacologic treatment. No predictive factors associated with the recurrence of anal CA were found.

**Fig 1:**
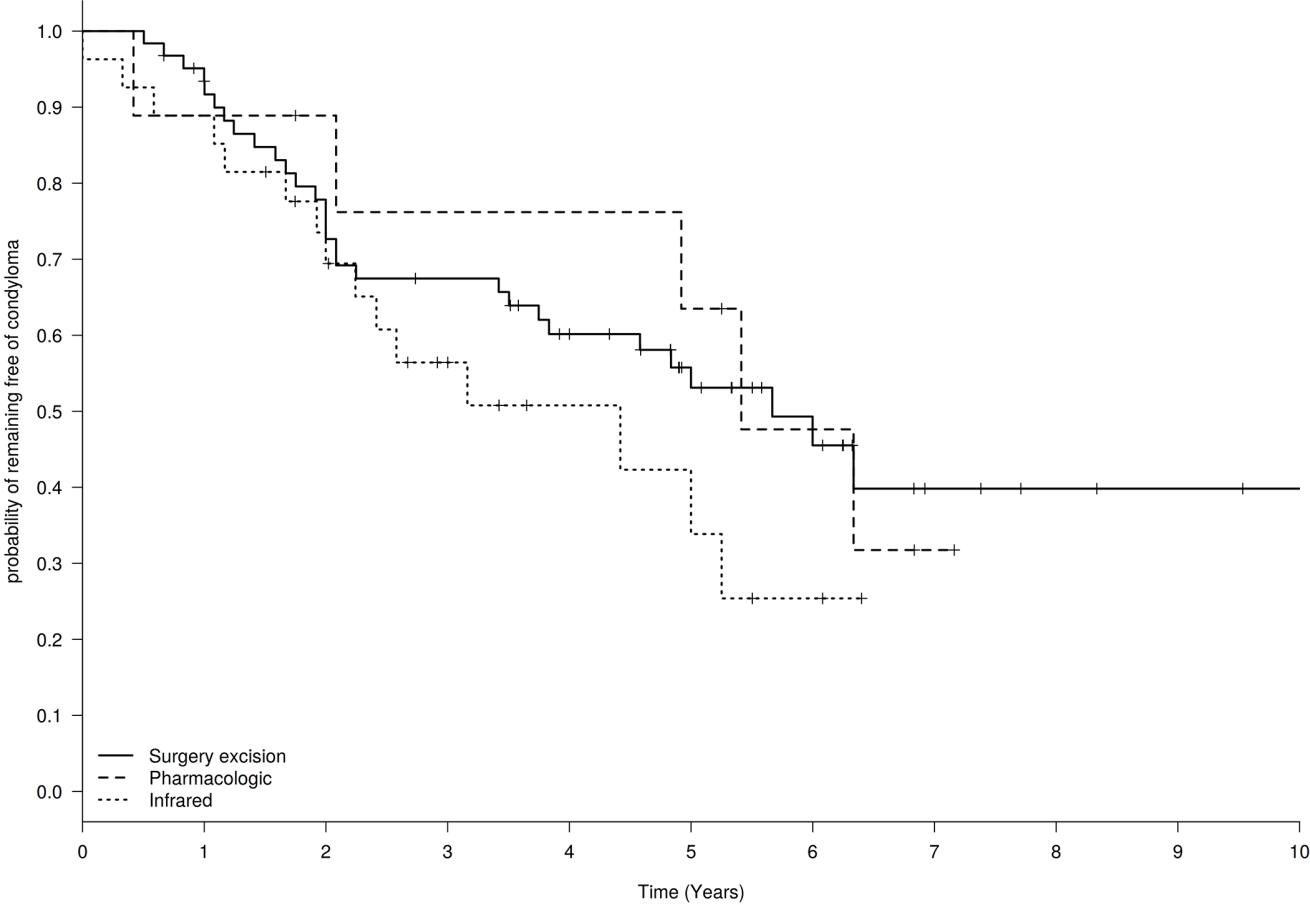
Actuarial probability (Kaplan–Meier curve) of remaining free of anal condylomata in HIV-infected men according to the different treatments performed: Electrosurgery excision (65 patients), infrared (27 patients) and imiquimod (9 patients).

After up to 10 years of follow-up, the overall cumulative recurrence rate was 49.5% (51 recurrence in 103 patients treated, 95%CI: 40–59%). The rate (range) of recurrence was 13.6 (10.2; 17.9) per 100 person-years. By treatments, the cumulative recurrence rate was 46% (30 patients, 95%CI: 35–58%) with electrosurgery excision, 56% (15 patients, 95%CI: 37–72%) with IRC, 56% (5 patients, 95%CI: 27–81%) with pharmacologic treatment and 50% (1 patient, 95%CI: 1–91%) with cryotherapy. The actuarial probability (Kaplan-Meier curve) of remaining free of anal CA by the different treatments is shown in Fig 1. The median time of recurrence was 4.0, 2.6, 5.3 and 5.7 years for electrosurgery excision, IRC, pharmacologic treatment and cryotherapy, respectively. A *post-hoc* analysis stratified by sexual behavior (men who have sex with men–MSM- or men who have sex with women–MSW-) was performed in patients who suffered either electrosurgery excision or infrared coagulation. No differences were observed.

**Fig 2:**
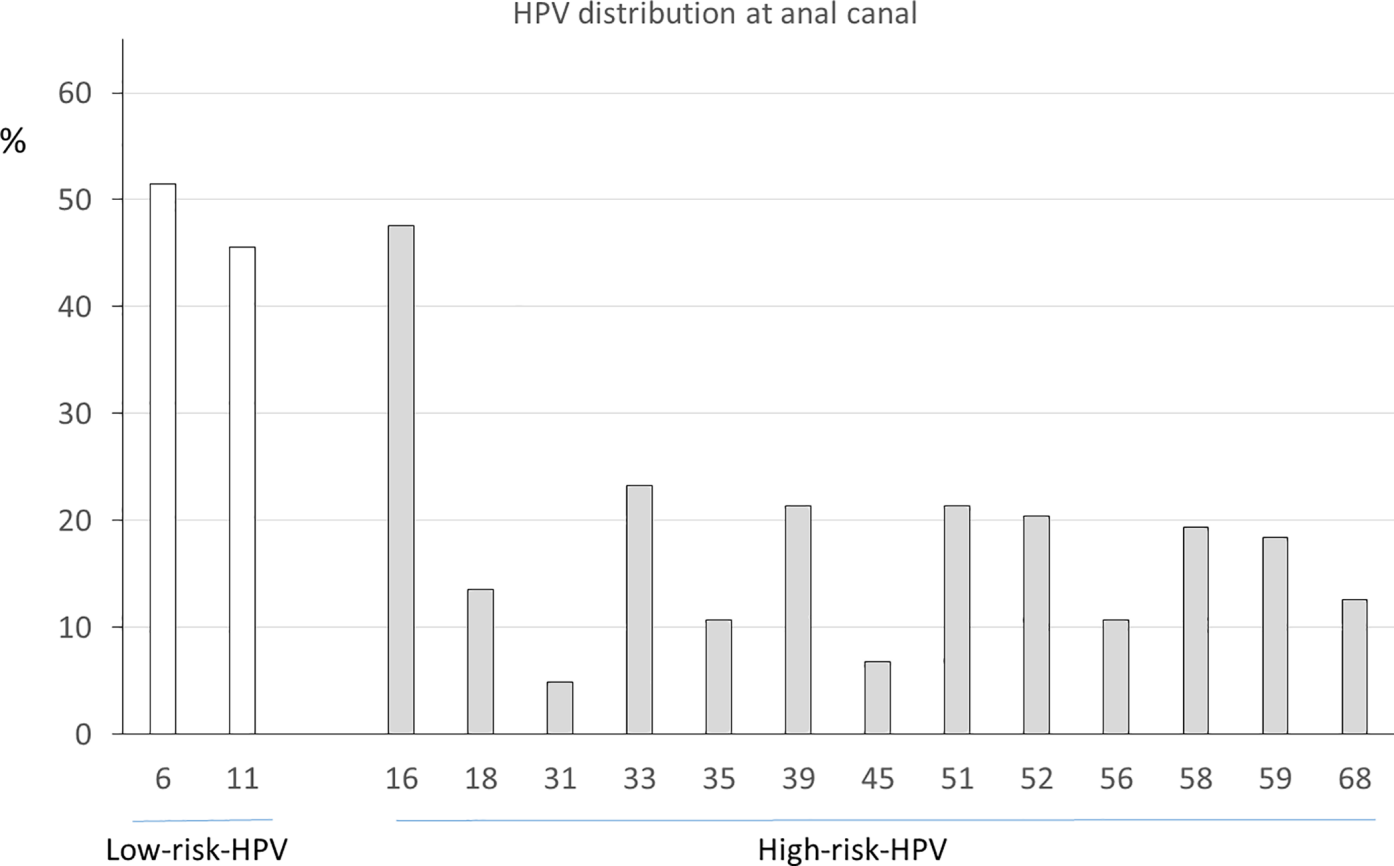
HPV genotypes distribution at the visit of anal condylomata diagnosis.

Table 2 shows the anal canal cytological results at the diagnosis of anal CA. Respect to SIL in anal canal, similar results were found between MSM and MSW. Fig 2 depicts the HPV genotypes distribution involved at CA diagnosis. All patients with anal CA had an HPV infection at anal canal with 2 or more HPV genotypes. The most predominant genotypes were HPV-6 (52%, 53/103, 95%CI: 42–61%), HPV-11 (46%, 47/103, 95%CI: 36–55%) and HPV-16 (48%, 49/103, 95%CI: 38–57%). The cumulative prevalence of genotypes 6 and 11 was 97% (100/103, 95%CI: 92–99%) in cytological samples from anal canal previous to receive the treatment for anal condylomata, therefore, HPV-6 or HPV-11 was not detected in only 3 patients. Likewise, it is noteworthy that cumulative prevalence of genotypes 16 and 18 was 61% (63/103, 95%CI: 52–70%), and the cumulative prevalence taking into account all high-risk HPV genotypes was of 100%. Respect to the other HPV genotypes involved in the new vaccine, the prevalence were 14% HPV-18 (14/103, 95%CI: 8–22%), 5% HPV-31 (5/103, 95%CI: 2–11%), 23% HPV-33 (24/103, 95%CI: 16–32%), 7% HPV-45 (7/103, 95%CI: 3–13%), 20% HPV-52 (21/103, 95%CI: 14–29%), and 19% HPV-58 (20/103, 95%CI: 13–28%).

**Table 2:** Anal canal cytological findings of HIV-infected men at the visit of condyloma diagnosis.

Anal canal cytology					
		Normal	ASCUS	LSIL	HSIL
**WHOLE POPULATION (n = 103)**					
**Electrosurgery excision**	n = 65	6 (9%)	11 (17%)	40 (62%)	8 (12%)
**Infrared**	n = 27	3 (11%)	4 (15%)	16 (59%)	4 (15%)
**Pharmacologic**	n = 9	4 (44%)	1 (11%)	4 (44%)	—
**Cryotherapy**	n = 2	1 (50%)	—	1 (50%)	—
	**total**	14 (14%)	16 (16%)	61 (59%)	12 (12%)
**MSM (n = 91)**					
**Electrosurgery excision**	n = 59	6 (10%)	11 (19%)	35 (59%)	7 (12%)
**Infrared**	n = 24	3 (13%)	3 (13%)	14 (58%)	4 (17%)
**Pharmacologic**	n = 6	2 (33%)	1 (17%)	3 (50%)	—
**Cryotherapy**	n = 2	1 (50%)	—	1 (50%)	—
	**total**	12 (13%)	15 (17%)	53 (58%)	11 (12%)
**MSW (n = 12)**					
**Electrosurgery excision**	n = 6	—	—	5 (83%)	1 (17%)
**Infrared**	n = 3	—	1 (33%)	2 (67%)	—
**Pharmacologic**	n = 3	2 (67%)	—	1 (33%)	—
**Cryotherapy**	n = 0	—	—	—	—
	**total**	2 (17%)	1 (8%)	8 (67%)	1 (8%)

### Adverse events related to procedures

Pain was the main adverse event reported by patients and related to treatments [7 (11%) patients after surgical excision, 2 (7%) patients after IRC and 1 (11%) after imiquimod]. Three (5%) patients had a stenosis develop in anal canal and 1 (2%) had an abscess in the surgical scar area after the electrosurgical excision. All patients treated with imiquimod had skin irritation or a burning sensation. No adverse event related with the obtaining of sample from anal canal for cytology and HPV assessment was gathered.

## Discussion

The aim of this study was not to compare the effectiveness of different treatments for anal condyloma in HIV-1-infected men, as treatment depended on factors such as the number of condyloma, size of affected area, location and characteristics of the patients. Nevertheless, to our knowledge, this is the first time that the effectiveness of different treatments (electrosurgery, infrared coagulation and imiquimod) for the treatment of anal CA in HIV-1-infected men (MSW and MSM) is evaluated during a long period of time. In spite of an excellent observed effectiveness at 3 months (no recurrence) and at one year after treatment (on 10% of recurrence), a high rate of CA recurrence was found when the follow-up was up to 10 years treatment (on 50% of recurrence). These recurrence rates were unrelated to treatment type. However, our descriptive results suggest that HIV-1-infected men treated for anal CA are a population with a high probability of suffering a recurrence, independent of the treatment used.

The decision to propose one of the treatments available in our hospital was mainly based on the characteristics of CAs (number, area affected…) and doctor’s experience [17]. Surgical excision was performed in over 60% of the patients. This data reflects that large condylomata, affecting internal and external area of anal canal, were the frequent among our HIV-1-infected men. Although complications of surgical excision were low, these were not exhaustively collected in our study. Consequently, they should not to be ignored. Ablation with IRC and pharmacological treatment (imiquimod) were offered to patients with smaller anal condylomata in comparison to patients treated with electrosurgery excision. Although the effectiveness of treatments should not be compared, the recurrence rate during the follow-up in each group approximated 50% of the treated patients. Moreover, no predictive factors (such as HPV infection) were associated with the recurrence of anal condyloma. In front of the lack of efficacy clinical trials [6], our results could suggest that an ideal treatment for all types of anal CA is currently not available, and to personalize the treatment, that is, to select the treatment based on number, localization, area affected and surgeon’s experience could be an acceptable option.

Anal CA often harbor high-grade intraepithelial neoplasia and squamous cell cancer, mainly in MSM [5,18]. Similarly, data on histopathological findings in anogenital condylomata showed a high rate (greater than 35% in MSM) of the coexistence the high-grade anal intraepithelial neoplasia within surgically excised warts [19]. We found that the coexistence of both pathologies, anal condyloma and anal canal cytological LSIL and HSIL (obtained prior to be treating the CA) was 70%. This figure was 86% if ASCUS is considered. The co-existence of an apparently benign lesions and a pre-cancerous lesion is unsettling. No patient evolved to cancer during the 10 years of study period. An explanation to this finding could be that these patients are involved in a screening program for anal cancer (CARH·MEN cohort) and this screening program for detection and treatment of anal intraepithelial neoplasia has demonstrated to be effective (S. Videla, unpublished data).

Low-risk HPV genotypes 6 and 11 are associated with the development of anogenital condylomata [20–22]. These genotypes were detected in practically in all patients. However, given that the long median time of recurrence (over 3 years), recurrence of anal CA may be due to a new infection, recurrence at the same site, or development of a metachronous lesion. In HIV-1-infected patients it is common to have a multiple infections with several HPV types including high-risk types that is independent of immune status [23,24]. In our study, a 70% of patients were infected by HPV-16 or HPV-18. If, besides, it is taken into account the rest of high-oncogenic risk HPV genotypes, all patients of our study were infected for high-risk oncogenic genotypes. In consequence, HIV-1-infected men with CA are a population with a high risk to develop anal squamous cancer. Therefore, a special attention in our clinical practice has to be taken into account with this population, HIV-1-infected men and diagnosis of anal condyloma.

Our study is subject to several limitations. Its observational retrospective design (e.g. the exact number of anal CAs per patient was not systematically gathered in the medical file, and therefore, this information is not provided) and the population analyzed (from a single geographical site and HIV-1-infected patients without a medical history of anal CA) could lead us to underestimate or overestimate the generalizability of the results beyond the population and conditions studied. As above-mentioned, the treatments were offered depending on the characteristics of the lesion/s, which could lead us to underestimate or overestimate the generalizability of the results beyond our clinical guidance protocol (treatment used depend on the characteristics of condyloma). In the case of imiquimod treatment, the compliance and its correct application cannot be guaranteed. The sample size could also lead us to underestimate or overestimate the generalizability of the results. HRA was only performed in patients with an abnormal anal cytology result; hence CA in the anal canal could be underdiagnosed. Likewise, we cannot know if the CA recurrence was related to new infection.

In summary, our results indicate that HIV-1-infected men, along their life, have a high rate of anal CA recurrence after physically ablative (electrosurgery, infrared) and pharmacological treatment (imiquimod). Randomized clinical trials are needed to best understand which the best treatment is. Furthermore, the high rate of the co-existence of CA and dysplasia at anal canal and the high percentage of high-risk HPV reinforce that, in clinical practice, the goal to manage the anal condylomata is to prevent the recurrence and to carry on with careful clinical follow-up of HIV-1-infected men. This population of HIV-1-infected men with CA are at risk of developing an anal cancer related to high-risk HPV genotypes.
